# Global trends and thematic evolution in online consultation with digital health technologies: Implications for future implementation and policy development

**DOI:** 10.1371/journal.pdig.0001779

**Published:** 2026-10-29

**Authors:** Jing Liu, Xiaoqian Gong, Tingting Yang, Ruoxin Xu, Liming Tan, Huizi Ye

**Affiliations:** 1 Office of Operations Management, Yuebei People’s Hospital Affiliated to Shantou University Medical College, Shaoguan, China; 2 Hospital Office, Yuebei People’s Hospital Affiliated to Shantou University Medical College, Shaoguan, China; 3 Science Research Office, Yuebei People’s Hospital Affiliated to Shantou University Medical College, Shaoguan, China; Instituto Politécnico Nacional Escuela Superior de Medicina: Instituto Politecnico Nacional Escuela Superior de Medicina, MEXICO

## Abstract

Online consultation has become a mainstream element of digital health within telemedicine, seeing rapid growth during the COVID-19 pandemic. Video, telephone, and secure messaging are extensively used for clinical advice, assessments, and follow-ups. However, the adoption of these technologies remains inconsistent, particularly in regions with limited infrastructure and workforce capacity. Key challenges include patient engagement, integration into clinical workflows, and the efficient allocation of resources. This bibliometric analysis covers 2,426 English-language journal articles indexed in the Science Citation Index Expanded (SCI-Expanded) within the Web of Science Core Collection (WoSCC) (1990–2025). Utilizing VOSviewer and the bibliometrix package, we mapped publication growth, international collaborations, keyword co-occurrence, and thematic evolution, with country-specific trajectories examined for the leading contributors. Countries were ranked via full counting, and joinpoint regression identified inflection points in annual publication output. Publication output grew steadily over the past 35 years, accelerating significantly after 2018, with a sharp peak from 2019 to 2022. The United States, England, China, Canada, Australia, and Germany were the main contributors and central hubs in international collaboration. The thematic focus shifted from early feasibility studies to service management, quality and outcome evaluation, barriers to implementation, equity considerations, and post-pandemic integration into standard care pathways. Country-specific trends were evident: the United States focused on telemedicine models and reimbursement mechanisms, England concentrated on primary care and general practice, and China experienced rapid growth in telehealth research post-2018. The literature on online consultation has evolved from proof-of-concept studies to a system-wide focus on management, evaluation, and large-scale implementation. These variations reflect differences in health system organization, regulation, reimbursement policies, and digital readiness across countries. Long-term reimbursement strategies, interoperable digital infrastructures, and equity-oriented service design may support the sustainable implementation of online consultation services.

## Author summary

In this study, we examine the growing role of online consultations in digital health, particularly within telemedicine. By analysing over 2,400 research articles from 1990 to 2025, we explore how online consultations have become an important service model in healthcare worldwide. The study also highlights differences in how various countries have adopted and integrated these services. Despite widespread growth, there are still challenges, particularly in areas with limited resources, such as infrastructure and workforce capacity. Our findings indicate that sustainable online consultation services may require long-term funding models, interoperable digital systems, and service designs that promote fairness and long-term sustainability. This research is vital for healthcare leaders and policymakers, offering guidance on how to improve online consultation services and make them accessible to a wider range of patients, particularly in the post-pandemic world.

## Introduction

### Background

Internet-based health care encompasses a range of services enabled by the internet and information and communication technologies (ICT) that complement conventional care pathways. Within this broader field, eHealth refers to ICT-supported health services, mHealth to services delivered through mobile and wearable devices, and telemedicine to clinical care provided over distance. Online consultation specifically refers to internet-mediated encounters in which patients and clinicians exchange advice, triage, diagnostic assessment, prescribing where permitted, and follow-up [1^,^^2^]. The World Health Organization places these services within the broader framework of digital health, emphasizing the secure and cost-effective use of ICT to support health care [3^,^^4^]. In China, this field is commonly operationalized through internet-based diagnosis and treatment, internet hospitals, and Internet Plus Healthcare. These services are generally linked to licensed clinicians and brick-and-mortar institutions and may form a closed-loop pathway from online consultation to offline testing or treatment and subsequent online follow-up [5].

Accelerated by the COVID-19 pandemic, online consultation has expanded from pilot use to routine delivery through both synchronous channels, such as video and telephone, and asynchronous channels, such as secure messaging and patient portals. Previous studies suggest potential benefits for access, continuity, and response time [6–8].

However, its implementation remains constrained by factors such as digital literacy, resource allocation, and coordination between online and offline services. Qiu et al [9] found that older adults may encounter difficulties related to device operation and limited digital competence when using internet-based health services. Li et al [10] reported that demand for online consultation may be concentrated among a small number of highly reputable physicians, reflecting an uneven distribution of resources across online platforms. Chen et al [11] further showed that, although internet-based health care can help maintain health service provision, stronger coordination mechanisms between online services and brick-and-mortar institutions are still needed.

Bibliometric analysis can quantify publication output and visualize the intellectual structure and temporal evolution of a research field [12]. Previous bibliometric and science mapping studies have profiled telemedicine, eHealth and digital health by describing publication growth, international collaboration networks and keyword co-occurrence patterns. However, the existing literature is relatively fragmented, with many studies focusing on particular countries, clinical specialties, user populations, or pandemic-related periods. For example, Ivanitskaya et al [13], examined the multidisciplinary development of eHealth research and its increasing interdisciplinary integration, whereas Maugeri et al [14] combined bibliometric methods with web search data to assess changes in public and academic interest in telemedicine between 2017 and 2022. Other studies have focused on specific settings or populations, including barriers and facilitators to real-time home-based online consultations [15], the experiences of patients with mental health conditions receiving remote consultations in primary care [16], and the impact of COVID-19 on the use of telemedicine in mental health care systems [17]. Jiang et al [18] further reviewed recommendation approaches for online medical consultation services, while Campbell et al [19] evaluated the effects of virtual consultations on the quality of primary care. Nevertheless, for online consultation as a distinct digital health service model, there remains a lack of systematic analyses that simultaneously integrate long-term publication trends, international collaboration structures, thematic evolution, and the research trajectories of leading countries.

To address these gaps, a unified SCI-Expanded corpus spanning from 1990 to 2025 (comprising 2,426 records) was constructed, with standardized terminology for online medical consultation. Descriptive indicators were integrated with VOSviewer and bibliometrix to analyze trends in annual publications, country-level collaboration networks, institutional affiliations, keyword clustering, thematic evolution, and emerging research areas. Countries were ranked using full counting, and the top six contributors were selected for comparative analysis. Joinpoint regression was applied to segment the timeline into early, middle, and recent periods, enabling the examination of country-specific research trajectories. Cross-national differences were then analyzed in the context of policy and infrastructure factors, providing a more nuanced interpretation of these variations.

### Objectives

This study maps the global knowledge structure and thematic evolution of online consultation research (1990–2025) using bibliometric and science-mapping methods. We further compare thematic trajectories across the top six producing countries and interpret differences in relation to policy and infrastructure contexts.

Compare annual trends of research articles to assess topic maturation and diffusion, and detect time lags between the emergence of active areas and subsequent publication growth. We asked the following question: How has online consultation scholarship developed over time? (Research question [RQ1])

We construct keyword co-occurrence networks and apply network clustering to delineate coherent themes and track how these configurations are reorganised over time. Changes in high-frequency terms and trending topics are used to summarise shifts in attention. On which scientific fields and knowledge bases underpin online-consultation research, and how have these foundations and associated topic emphases evolved across time windows? (RQ2)

Focusing on the six most productive countries, we compare publication trajectories and collaboration structures, and then repeat the keyword co-occurrence and clustering procedures within each country-specific subset to derive comparable thematic profiles. We further contrast thematic trajectories and emerging topics across countries. How do leading countries differ in their thematic structures and trajectories, and which contextual factors plausibly account for these differences? (RQ3)

## Materials and methods

### Ethics considerations

Ethics approval was not required because this study is a retrospective bibliometric analysis of published literature and did not involve human participants or identifiable personal data.

### Data sources and search strategy

Fig 1 presents a PRISMA 2020-adapted flow diagram adapted for bibliometric dataset construction for study identification and selection, and the full search strategy is reported accordingly [20]. The bibliometric workflow followed established guidance proposed by Cobo et al [21]. Because online consultation research is inherently interdisciplinary and bibliometric mapping is sensitive to differences in database coverage and metadata structure, we conducted preliminary searches in SCI-Expanded, Scopus, and PubMed to compare retrieval performance, DOI availability, and metadata completeness. SCI-Expanded was retained as the primary data source because it provided structured cited-reference, affiliation, country/region, and keyword-related fields compatible with the planned bibliometric workflow [22]. The supplementary database comparison, including retrieval volume, DOI availability, DOI overlap, and database-specific search strategies, is provided in Tables C-F in S1 Appendix.

**Fig 1:**
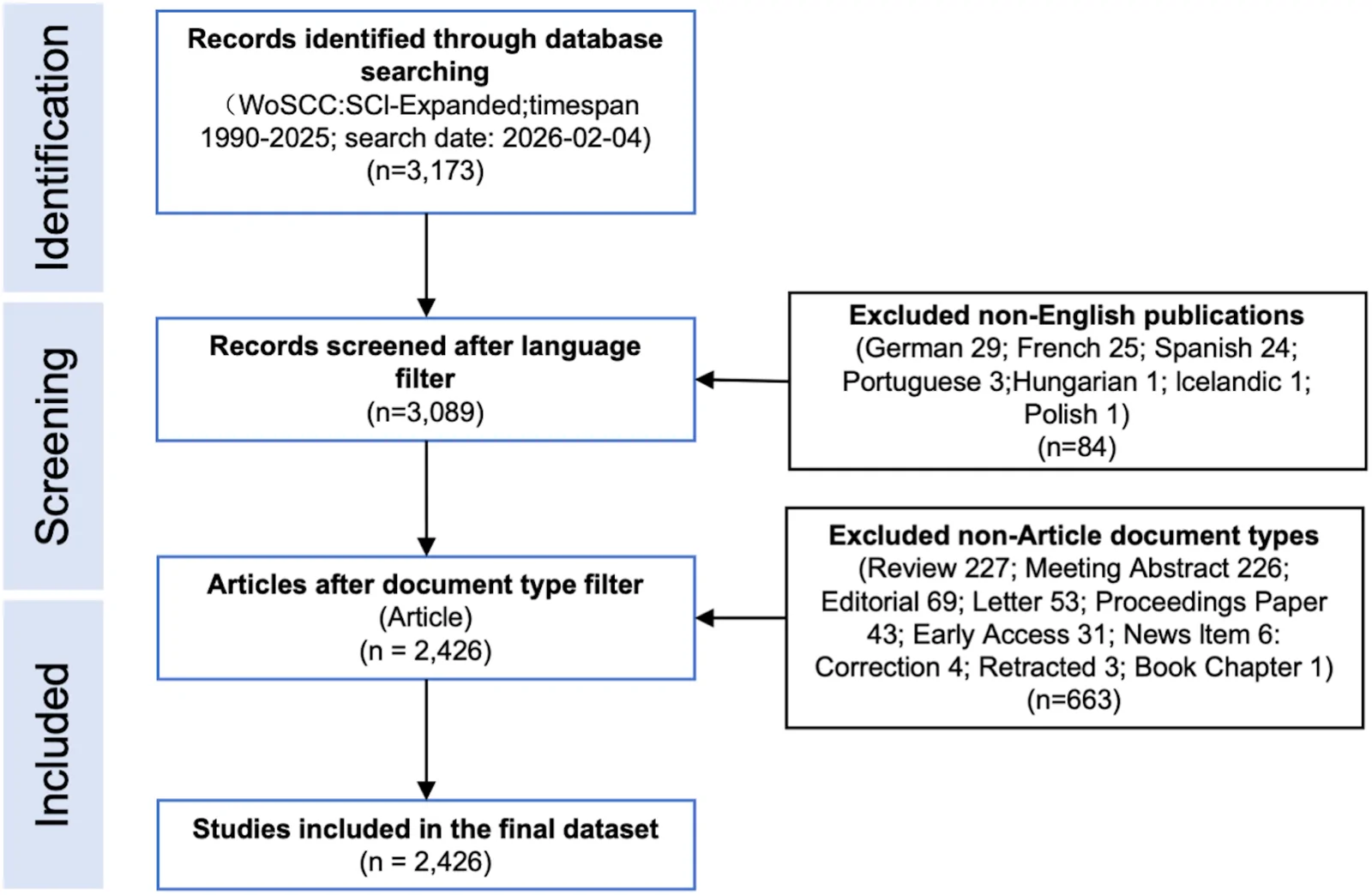
Trends and selection in online consultation research. PRISMA flow diagram of online consultation publications study selection. SCI-Expanded: Science Citation Index Expanded.

The search strategy was jointly developed by a medical librarian, domain experts in internet-based health care, and specialists in medical informatics. We searched the Science Citation Index Expanded (SCI-Expanded) within the WoSCC for records published between January 1, 1990 and December 31, 2025. To capture online consultation research, we searched the Topic field (title, abstract, and author keywords) and operationalized the query around two core concept blocks: (1) health care and patient-clinician consultation terms; and (2) online consultation term. Terms within and across concept blocks were combined using Boolean operators. To minimize variability due to database updates, records were retrieved and exported on February 4, 2026 (Full Record and Cited References; plain-text format). The exact SCI-Expanded search query and sequential filtering workflow are provided in Tables A-B in S1 Appendix. To improve reproducibility,Fs a metadata-level derivative dataset containing the included records and key bibliographic fields was deposited as S1 Data, as described in the Data Availability Statement.

### Eligibility and exclusions

Records indexed in SCI-Expanded within the prespecified time window and classified as English-language Article were eligible for inclusion. We excluded records outside the time window, non-English publications, and document types other than Article. To enhance topical relevance, we further removed clearly nonmedical or non-health service records by applying an exclusion filter in the Topic field (e.g., TS=(legal OR law OR marketing OR business OR “academic advising”)). Detailed record counts for the initial retrieval, language filter, document-type filter, and final analytical corpus are reported in Table B in S1 Appendix. Duplicate records and outdated versions were identified using the WoS unique identifier (UT). When multiple versions were available, we retained the most complete and final record for verification and analysis.

During screening, a pilot set of 50 records was independently reviewed by two assessors (TT and RX) to assess screening consistency. Any disagreements arising during the screening process were resolved by adjudication from a third reviewer (HZ). The bibliometric screening was completed on February 5, 2026.

### Data analyses

#### Data cleaning.

To ensure consistency across tools used for scientific mapping, we imported the plain-text export from SCI-Expanded within the WoSCC into RStudio (R environment) for deduplication and standardization. Data import and wrangling were performed primarily using the readr package to parse TXT files, dplyr for record filtering, grouped summaries, and counting, and stringr for text normalization. For keyword processing, we developed a keyword-merging dictionary to consolidate synonyms, singular/plural forms, spelling variants, hyphenation differences, abbreviations, and clearly synonymous expressions, thereby reducing conceptual fragmentation that could bias co-occurrence patterns and topic evolution analyses. Broader conceptual categories were not merged. Before consolidation, the analytical keyword file contained 554 original keyword labels and 11,329 keyword occurrences. The keyword-merging dictionary mapped 151 variant labels to standardized terms, affecting 2,522 keyword occurrences, or 22.3% of all keyword occurrences. After consolidation, the number of unique keyword labels was reduced from 554 to 472, representing a reduction of 82 labels, or 14.8%. The quantitative summary, sensitivity comparison before and after keyword consolidation, and complete keyword-merging dictionary are provided in Tables A-D in S2 Appendix. To partially account for differences in country-specific corpus size, we conducted a sensitivity analysis using standardized keyword frequencies; for each of the six countries, the top 10 standardized keywords were extracted, and keyword occurrences were normalized per 100 country-specific records. The normalized keyword-frequency results are reported in Table A in S6 Appendix.

#### Country selection and comparative design.

We used a full-counting approach to aggregate author affiliations at the country level. Country/region information was taken from the WoS Countries/Regions field. Based on the final corpus, we prespecified the six most productive countries/regions for in-depth comparison: the US (n = 891), England (n = 301), China (n = 176), Canada (n = 150), Australia (n = 128), and Germany (n = 102). This ranking was consistent with the stacked area plot of annual country-level contributions.

#### Bibliometric approach.

After constructing the corpus and completing deduplication and standardised cleaning, we applied a multi-tool bibliometric workflow. VOSviewer (v1.6.20, Leiden University) [23] was used to map author and institutional collaboration networks, perform keyword co-occurrence clustering, and run journal bibliographic coupling analyses. Networks used association-strength normalization; node size represents publication output or keyword frequency, and edge weights represent collaboration or co-occurrence strength. For author analyses, we applied a threshold informed by Price’s approach to identifying core authors [24] and generated corresponding visual maps. For institutional collaboration, to improve stability and interpretability, we included only institutions with ≥5 publications (n = 252). Institutions were then classified by primary function (universities, hospitals, companies, and research institutes/government agencies), and cross-type collaboration intensity was summarised using a matrix-based representation. We used bibliometrix (R) [25] to examine thematic evolution, trending topics, and cross-country thematic structures, with topic clustering implemented using the Walktrap algorithm. The allocation of bibliometric tools to analytical functions is summarized in Table A in S3 Appendix. Joinpoint regression was applied to identify change points in annual publication trends and to estimate segmented trends. Candidate models with 0, 1, and 2 joinpoints were compared; detailed model-selection statistics, joinpoint locations with 95% confidence intervals, and segmented estimates are reported in Tables A-B in S5 Appendix. All R-based analyses were conducted in RStudio. Figures were subsequently refined for layout and annotation in GraphPad Prism 10 and Microsoft Excel.

## Results

### Temporal growth of online consultation scholarship

A total of 2,426 publications indexed in the SCI-Expanded from 1990 to 2025 were included for bibliometric analysis, covering a 35-year retrieval window. The field produced an average of 69.31 publications per year, with an average annual increase of 17.39 publications. Annual output exceeded 100 for the first time in 2020 (n = 197, 8.12%) and peaked in 2021 (n = 365). Although publication counts declined modestly during 2022–2025, they remained substantially higher than pre-2020 levels (Fig 2A). To address RQ1 and visualize long-term growth, we used a second-order polynomial as a descriptive fit for cumulative publications (R^2^ = 0.90). We further applied joinpoint regression to detect structural changes in annual publication trends. Candidate models with 0, 1, and 2 joinpoints were compared, and the final selected model contained two joinpoints. This model included 35 observations, 6 parameters, and 29 degrees of freedom, with SSE = 10,359.43, BIC = 6.2998, and WBIC = 6.4878. The two joinpoints were located in 2018 (95% CI: 2017–2019) and 2022 (95% CI: 2022–2022). The segmented slopes indicated three phases: a slow increase during 1991–2018 (slope = 1.87, SE = 0.17, P < 0.001), a rapid rise during 2018–2022 (slope = 82.99, SE = 4.81, P < 0.001), and a post-peak decline during 2022–2025 (slope = -49.83, SE = 4.81, P < 0.001). Full joinpoint model-selection statistics and segmented estimates are provided in Tables A-B in S5 Appendix. Taken together, the annual series and segmented estimates suggest an evolution from a prolonged low-baseline period to a pandemic-associated surge, followed by post-peak adjustment while publication activity remained at an elevated level compared with the pre-2020 period (Fig 2B).

**Fig 2:**
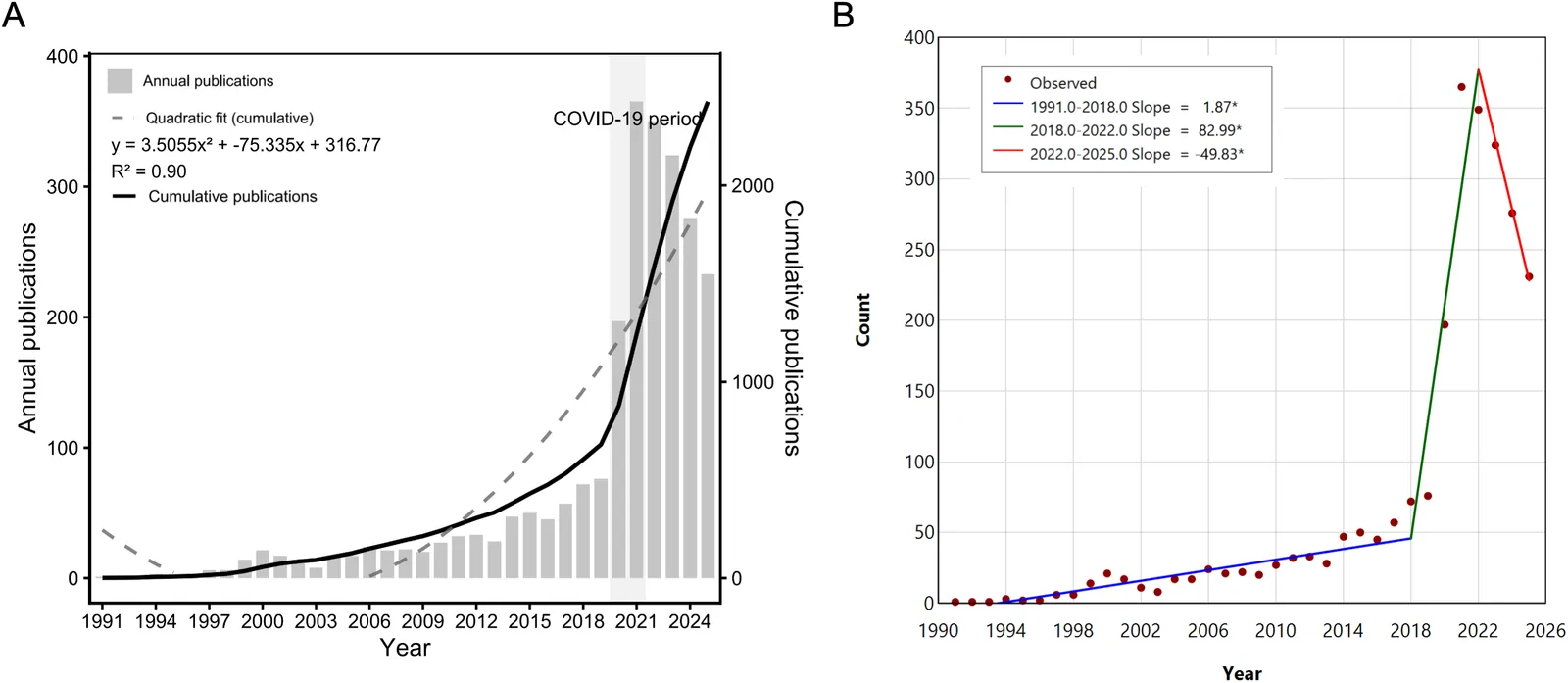
Temporal trends in publications on online consultation research. **(A)** Annual and cumulative numbers of publications from 1990 to 2025. Gray bars represent annual publications, the solid black line represents cumulative publications, and the dashed gray line represents the quadratic fit of cumulative publications. The shaded area indicates the COVID-19 period. **(B)** Joinpoint regression of annual publication output. Red points represent the observed annual publication counts, and the colored lines represent the fitted trends for 1991–2018, 2018–2022, and 2022–2025. The selected model identified two joinpoints: 2018 (95% CI 2017–2019) and 2022 (95% CI 2022–2022). Asterisks indicate statistically significant segment-specific slopes.

### Global landscape: Countries and collaboration

Among the 2,426 publications included from 1990 to 2025, country-level output was highly concentrated (Fig 3A). Of the top 10 most productive countries, the US contributed 891 publications (36.73%), followed by England (301, 12.41%), China (176, 7.25%), Canada (150, 6.18%), Australia (128, 5.28%), and Germany (102, 4.20%). These six countries accounted for 72.05% of the total literature, indicating that knowledge production in this field is dominated by a small group of high-output countries. Italy (98), the Netherlands (97), France (92), and Spain (76) also made significant contributors. The country collaboration network formed multiple clusters, with the US occupying the central position and maintaining strong collaborative ties with England, Canada, Australia, and China.

**Fig 3:**
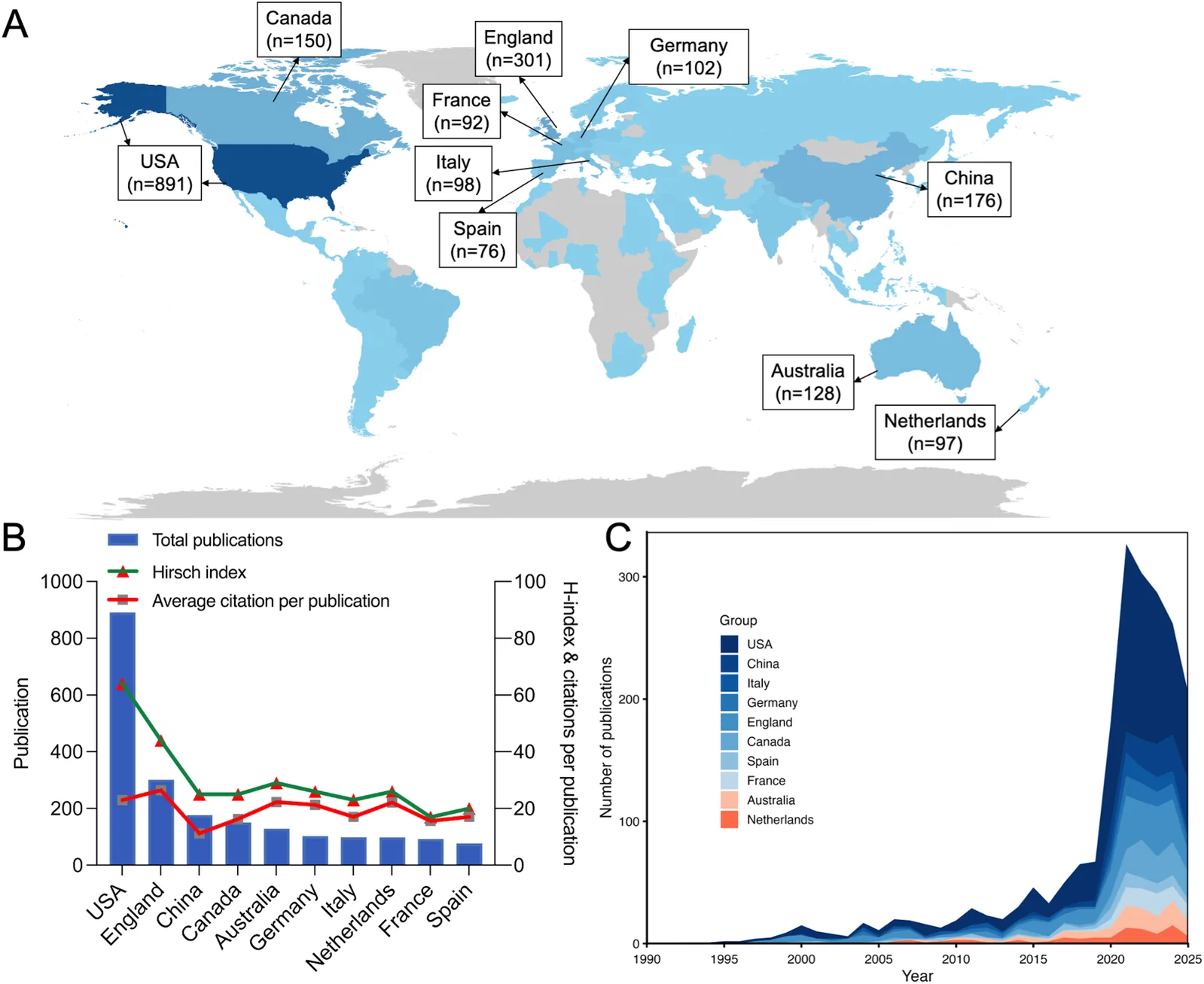
Scientific production and impact by country. **(A)** A global map of publication output, with callouts labeling the top 10 countries/regions by publication count **(n)**. The country boundary basemap used in Fig 3A was obtained from Natural Earth geographic data (https://www.naturalearthdata.com/). Natural Earth geographic data are released under a public domain license; terms of use are available at https://www.naturalearthdata.com/about/terms-of-use/. **(B)** Output and impact metrics for the top 10 countries/regions. Bars represent total publications, and lines represent the Hirsch index (H-index) and average citations per publication. **(C)** A stacked area chart of annual publications showing changes in country-level contributions from 1990 to 2025.

To further differentiate the nature of national contributions, we compared country-level impact indicators, including H-index and citations per paper (Fig 3B). There was a discrepancy between output volume and impact. The US led in productivity and had the highest H-index (H = 64), reflecting dominance driven by scale. England, despite publishing 301 papers, had the highest citations per paper (26.47), indicating a stronger impact per output. Australia produced 128 publications but maintained a relatively high H-index (H = 29), while the Netherlands achieved H = 26 with 97 publications. In contrast, China published 176 papers (H = 25) but had the lowest citations per paper (11.23), suggesting that its output growth has not yet translated into proportional impact.

We further examined the temporal distribution of publications among high-output countries using a stacked area chart (Fig 3C), which revealed heterogeneous trajectories of sustained engagement. Overall, contributions from these countries increased steadily after 2015, with accelerated growth in recent years. The USA, Italy, and Germany entered the field earlier (around 2008) and maintained relatively stable output, consistent with sustained investment and first-mover engagement. In contrast, China entered a more stable production phase after 2014 and has shown a sharper rise in recent years, reflecting the rapid increase in research attention to online consultation in China.

### Affiliations and organization types

Across the included publications, we identified 4,049 unique organizations from author affiliations. Of these, 252 organizations met the inclusion threshold (≥5 publications per organization) and were retained for visualization (see Table A in S4 Appendix). The institution-level co-authorship network derived from this threshold comprised five clusters and was dominated by two major collaboration blocs: North America and Europe-Australia with limited linkage to a small number of Asian nodes. This pattern indicates cross-regional collaboration but with clear dominance by high-output regions. In terms of productivity and network prominence, the top three institutions by publication count were Harvard Medical School (n = 76), the University of Toronto (n = 49), and the University of California, San Francisco (n = 45). By total link strength (TLS), the top three were the University of Toronto (TLS = 12,725), Harvard Medical School (TLS = 8,216), and the University of Oxford (TLS = 5,966). By total citations, the top three were Harvard Medical School (1,586 citations), the University of Pennsylvania (1,543 citations), and the University of Toronto (1,258 citations). Overall, more productive institutions tended to occupy more central positions within the collaboration network. Notably, although the University of Toronto ranked second in publication volume, it had the highest TLS, underscoring its hub-like role in inter-institutional collaboration (Fig 4A).

**Fig 4:**
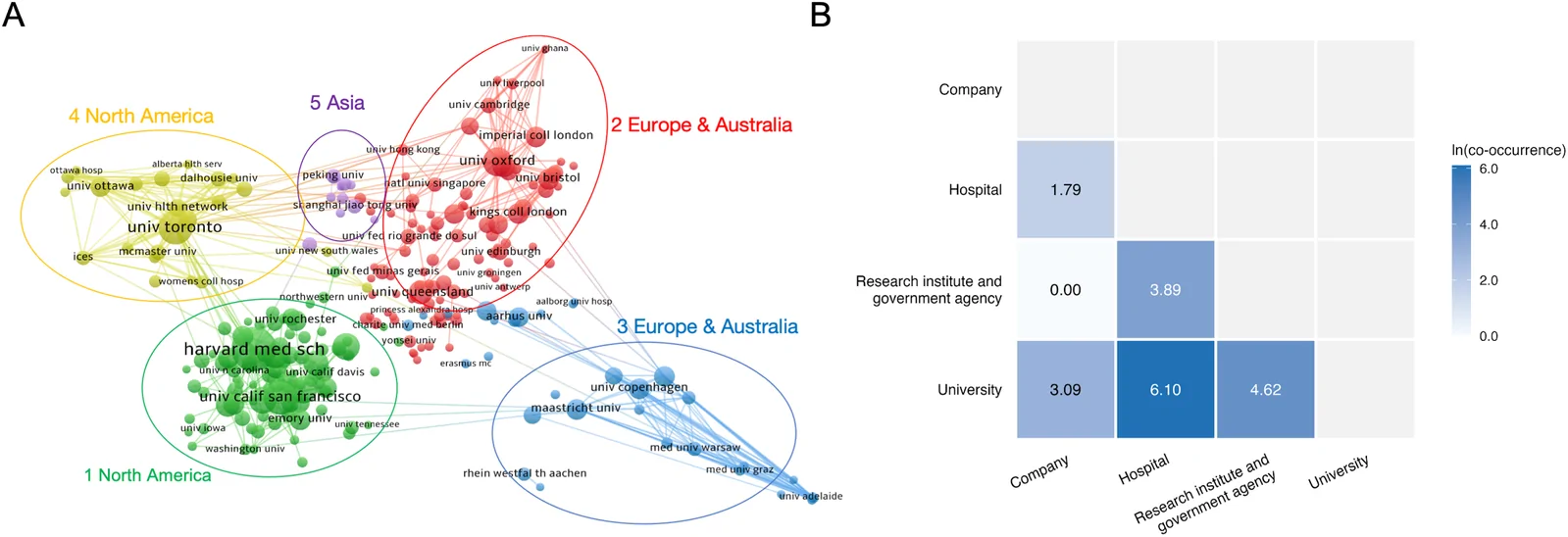
Institutional collaboration and cross-type collaboration intensity. **(A)** Co-authorship network of institutions with≥5 publications (n = 252). **(B)** Co-occurrence heatmap across organization type. Values are ln-transformed co-occurrence intensity; darker cells indicate stronger collaboration.

To characterize cross-sector collaboration, we classified 252 institutions into four categories by primary function: universities, hospitals, companies, and research institutes/government agencies. Regarding institutional contributions, 188 universities accounted for 2,153 publications (80.2%, mean 11.5 per university), 49 hospitals contributed 425 (15.8%, mean 8.7 per institution), 4 companies contributed 32 (1.2%, mean 8.0 per company), and 11 research institutes/government agencies contributed 76 (2.8%, mean 6.9 per organization). Since a single paper can be co-authored by multiple institutions, these counts are not mutually exclusive. We then constructed an institutional co-occurrence matrix, using the number of co-authored papers between each pair of institutional types as the co-occurrence intensity and applied a natural logarithm (ln) transformation for scale normalization (Fig 4B). Universities were the most active collaborators across sectors: university-hospital collaboration was the strongest (ln = 6.10), followed by university-research institute (ln = 4.62) and hospital-research institute (ln = 3.89) collaborations. In contrast, company-involved collaborations were relatively weak. The institutional composition further shows a high representation of clinically oriented providers and academic health centres (e.g., Harvard Medical School, Mayo Clinic, and Massachusetts General Hospital), suggesting a strong translational and public health orientation in the field.

### Author analysis

A total of 14,660 researchers contributed to the 2,426 publications included in this study. Author productivity followed a pronounced long-tail distribution, with most authors publishing only a few papers, while a small group of prolific authors accounted for a disproportionate share of the output. Following Price’s approach to identifying core authors, we set the threshold for core authorship at ≥3 publications. In total, 309 researchers (2.1%) met this criterion and collectively contributed 576 publications (23.87%) (Fig 5A).

**Fig 5:**
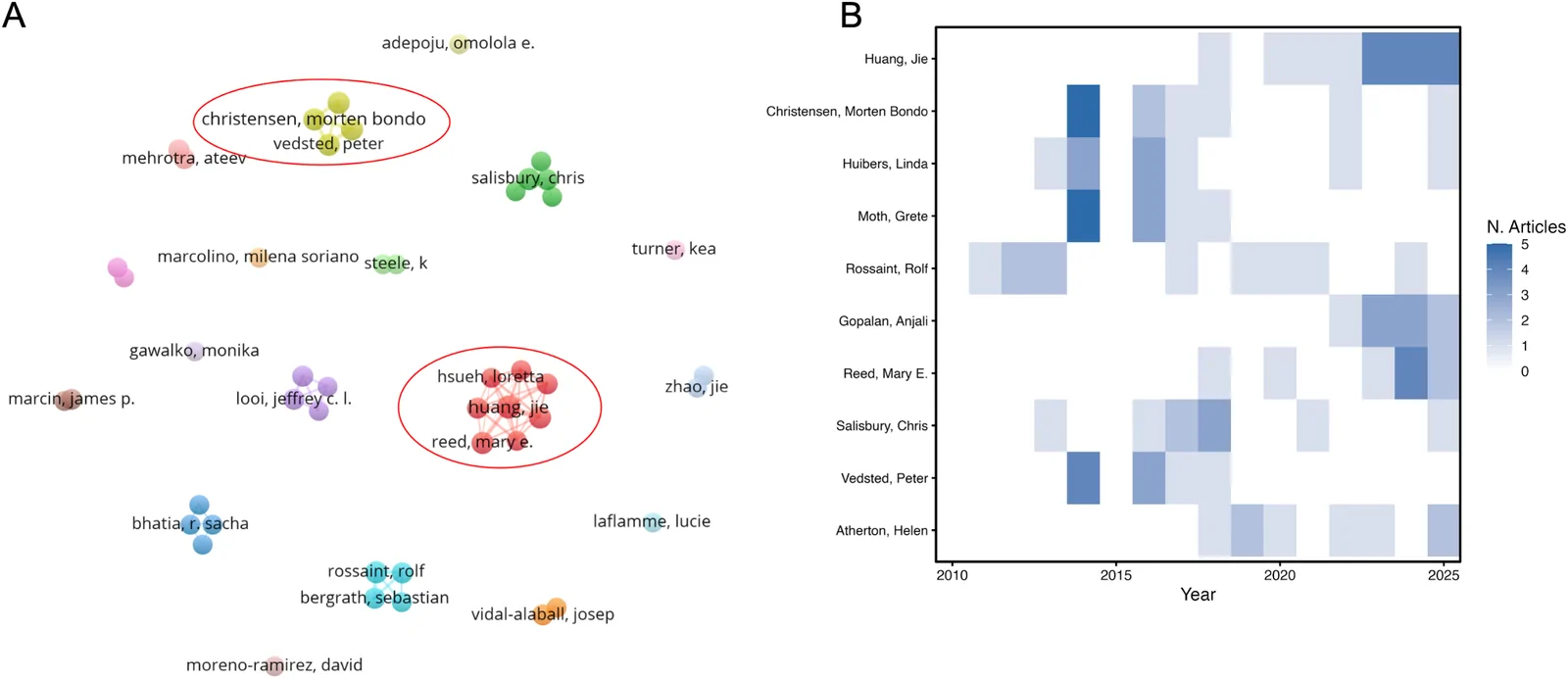
Co-authorship network of core authors and annual productivity heatmap. **(A)** Co-authorship network of core authors. Nodes represent authors, with node size proportional to publication output. Links indicate co-authorship, with thicker links representing stronger collaboration. **(B)** Annual publications of the top 10 most prolific authors from 2010 to 2025. The x-axis shows the publication year, and the y-axis lists the authors.

Among the most prolific authors, Jie Huang (Kaiser Permanente, USA) ranked first with 16 publications, followed by Morten Bondo Christensen (Aarhus University, Denmark) with 11 publications. Other highly productive contributors (≥10 publications) included Linda Huibers, Grete Moth, Mary E. Reed, and Rolf Rossaint (see Table B in S4 Appendix). The core-author co-authorship network showed dense within-cluster collaboration and comparatively limited cross-cluster links, indicating cohesive author communities. The largest subnetworks were centered on Jie Huang (e.g., Mary E. Reed and Anjali Gopalan) and on Morten Bondo Christensen (e.g., Linda Huibers, Grete Moth, and Peter Vedsted) (Fig 5B). To examine recent productivity dynamics, we summarized annual publications of the top 10 authors from 2010 to 2025. Output was episodic rather than continuous: a pronounced early peak occurred in 2014 (e.g., Christensen, Moth, and Vedsted), followed by a more dispersed phase, and a renewed concentration in 2023–2025, led by Jie Huang, Anjali Gopalan, and Mary E. Reed.

### Cross-country keyword similarity

To compare thematic trajectories and structural similarity across the six most productive countries/regions, we visualized trend-topic lifespan timelines and a keyword similarity matrix. In the lifespan timelines (Fig 6A), the active periods (Q1-Q3) and median years of each country’s top 10 trending topics showed consistent phase differentiation. Three inflection points-2011, 2018, and 2022-broadly partitioned research activity into three stages. Overall, topic activity increased substantially after 2018, and several countries exhibited a more recent-skewed concentration after 2022, suggesting a post COVID-19 structural realignment following pandemic-driven expansion. In terms of topic content, England remained anchored in primary care practice, the USA increasingly emphasized telemedicine alongside themes such as disparities and health care, China’s more recent topics clustered around platform-enabled provision, including information, internet hospital, and online consultation, Canada and Australia more prominently featured application-oriented themes such as teleconsultation, virtual care, and telepsychiatry, and Germany retained a stronger presence of implementation and specialty related themes in addition to telemedicine. To examine whether the country-specific keyword patterns were robust to large differences in corpus size, we supplemented the raw top-keyword comparison with normalized keyword frequencies per 100 records. The normalized analysis retained the same broad country-specific patterns, including the USA emphasis on telemedicine, telehealth, access, and health disparity; England’s primary-care and consultation-oriented profile; China’s internet-hospital and online-consultation profile; and the virtual-care, specialty, and implementation themes observed in Canada, Australia, and Germany. The normalized keyword-frequency results are provided in Table A in S6 Appendix.

**Fig 6:**
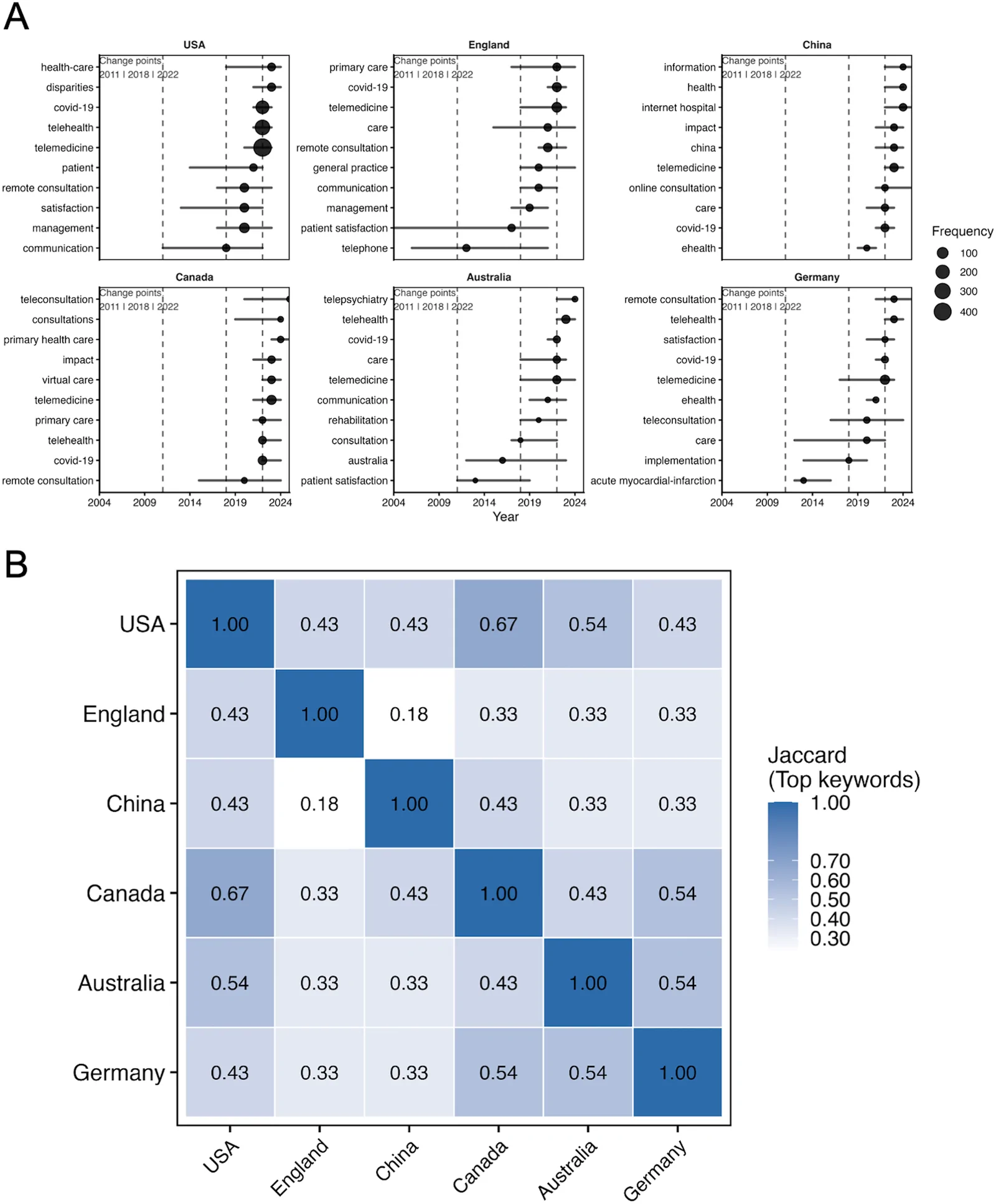
Trend-topic lifespan timelines and keyword similarity across six countries. **(A)** Lifespan timelines of the top-10 trend topics for the six most productive countries. Horizontal segments indicate the active period (Q1-Q3), dots indicate the median year, and dot size reflects frequency (vertical dashed lines indicate change points: 2011/2018/2022). **(B)** Pairwise Jaccard similarity matrix computed from each country’s standardized top-10 keyword set (darker shading indicates higher similarity; diagonal = 1.00).

Fig 6B presents the pairwise Jaccard similarity matrix based on each country’s top 10 standardized high-frequency keywords. Similarity values ranged from 0.18 to 0.67. The USA and Canada showed the highest similarity (Jaccard = 0.67), whereas England and China showed the lowest similarity (Jaccard = 0.18). Because top-keyword similarity is sensitive to the selected threshold, we conducted a supplementary analysis using the top 15 standardized keywords as a broader common threshold. Under the top-15 threshold, pairwise similarities ranged from 0.30 to 0.43; the supplementary top-15 Jaccard matrix is provided in Fig A in S6 Appendix. These results should be interpreted as descriptive indicators of keyword overlap rather than comprehensive measures of thematic convergence.

### Cross-country topic trajectories

To address RQ2, we used an alluvial (Sankey) visualization of keyword streams to characterize how themes shifted and were reconfigured over time across countries. The results indicate clear phase transitions. During 1991–2010, topics were dispersed across service delivery, telemedicine, communication, systems, effectiveness, and clinic-related themes, reflecting an early emphasis on feasibility and service context. During 2011–2018, themes became more consolidated, with nursing emerging as a central stream alongside parallel determinants, while peripheral expansion occurred toward diagnosis, programs, primary care practice, epidemiology, risk, and attitudes. During 2019–2022, management-oriented themes rose sharply and coupled with reliability, modeling, and epidemiology, signaling a shift from whether remote care can be delivered to how it can be managed and made methodologically robust. During 2023–2025, care and management remained dominant, while trials, teledentistry, follow-up, interviews, and challenges became more prominent, indicating increased attention to experimental evidence, longitudinal evaluation, specialty expansion, and systematic assessment of implementation barriers and user feedback (Fig 7).

**Fig 7:**
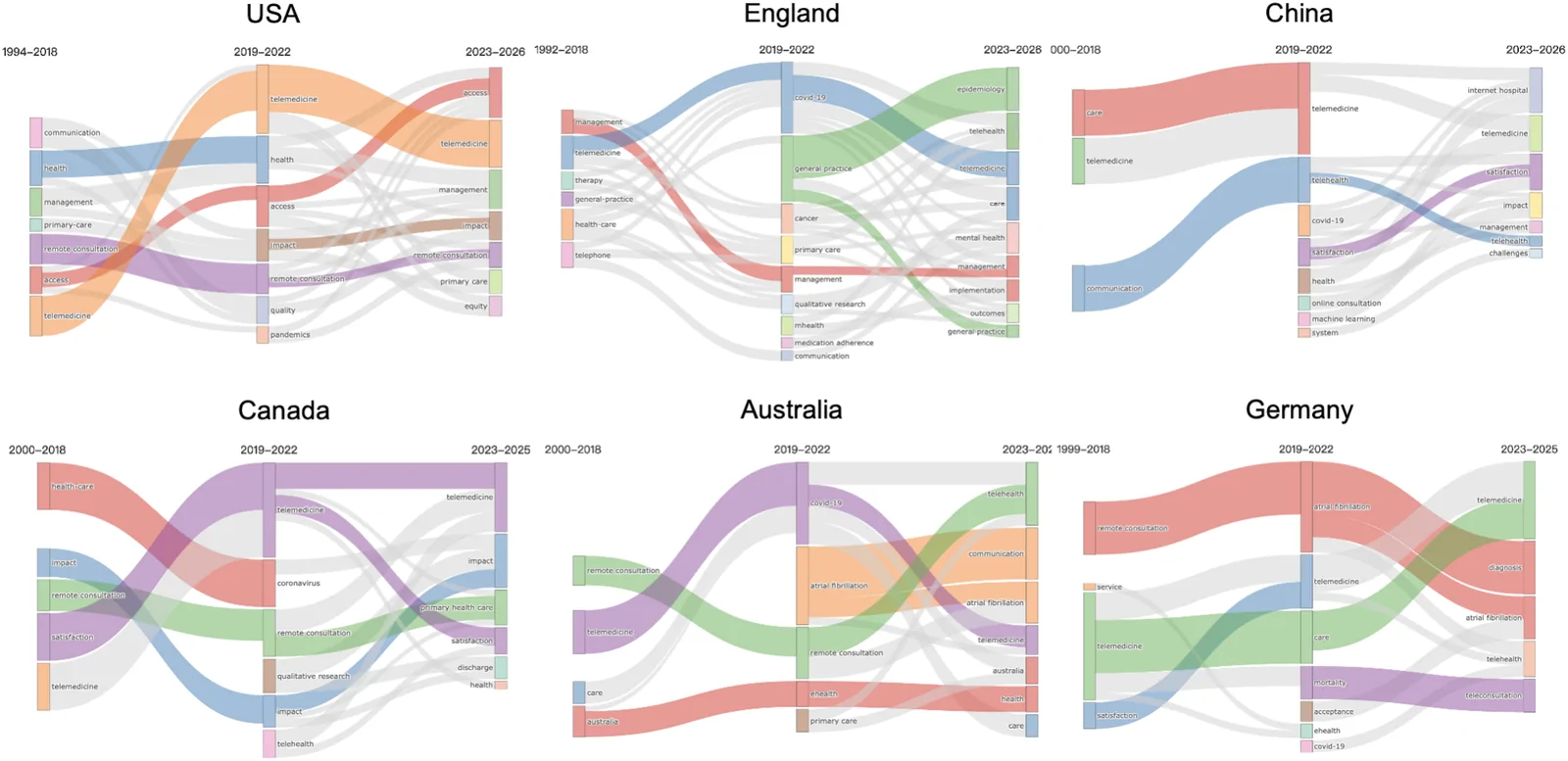
Temporal evolution of major keywords by country. Each panel shows one country and three time periods. Blocks are high-frequency keywords, flows link adjacent periods, indicating thematic continuity. Wider flows indicate higher relative weight, colors denote thematic streams.

Prior to 2018, research was largely organized around a telemedicine backbone, with foundational themes such as communication, access, primary care practice, health, and management. From 2019 to 2022, the thematic structure was heavily shaped by the COVID-19 context. The US retained health, telemedicine, and access as core themes, expanding into impact, quality, and pandemics. England was anchored in COVID-19 and general practice, with connections to management, cancer, primary care, and methodological topics. China exhibited parallel themes of care, telemedicine, and communication, with additional focus on telehealth, COVID-19, and patient satisfaction. Canada focused on telemedicine, coronavirus, and remote consultation. Australia highlighted COVID-19 and connected to atrial fibrillation, remote consultation, and health. Germany showed a dual-core focus on atrial fibrillation and telemedicine.

After 2023, the research focus shifted from pandemic-driven service expansion to routine adoption and outcome-oriented evaluation. The US increasingly emphasized access, management, impact, and equity. England pivoted to epidemiology, telehealth, implementation, outcomes, and mental health. China foregrounded internet hospitals and expanded into satisfaction, impact, management, telehealth, and challenges. Canada retained its telemedicine backbone while concentrating more on impact, primary care, satisfaction, and outcome-related themes (e.g., discharge/health). Australia remained telehealth-centered while strengthening communication and cardiovascular-specialty themes. Germany deepened from telemedicine to diagnosis and disease-specific themes (e.g., atrial fibrillation and teleconsultation).

Taken together, the shared cross-country trunk pathway suggests a common trajectory from telemedicine as a technical entry point, to rapid pandemic-associated expansion linked with primary care, system delivery, and patient experience themes (2019–2022), and then post-2023 differentiation towards access and equity (US), implementation and population health (England), internet hospitals and implementation challenges (China), primary care integration and outcomes (Canada), and deepened specialty applications (Australia and Germany).

### Trend topics

To address RQ3, we constructed country-specific keyword co-occurrence networks for the six most productive countries and applied community detection to identify thematic clusters. All six networks were built around a shared hub of telemedicine-related terms, forming a stable backbone linked to COVID-19 and care delivery themes. However, the network topology diverged across countries. The US and England exhibited denser networks with more clusters, suggesting broader thematic coverage and more extensive topic expansion. China also showed a multi-cluster structure, reflecting parallel platform service streams, while Canada, Australia, and Germany were more concentrated around a smaller set of core nodes, indicating more constrained thematic peripheries.

Country-specific emphases were evident in peripheral extensions. The US more strongly foregrounded public health, access, and health disparities. England reinforced a primary care context. China featured internet hospitals as a prominent platform-centered node. Canada highlighted the linkage between virtual care and primary care. Australia extended toward applied scenarios such as telepsychiatry and rehabilitation, while Germany showed stronger connections to system-level implementation themes and selected disease contexts. Taken together, these findings indicate a shift from whether remote care is possible to how online consultation can deliver measurable value. Future work should specify the health-system settings, target populations, and delivery models most conducive to sustainable impact (Fig 8).

**Fig 8:**
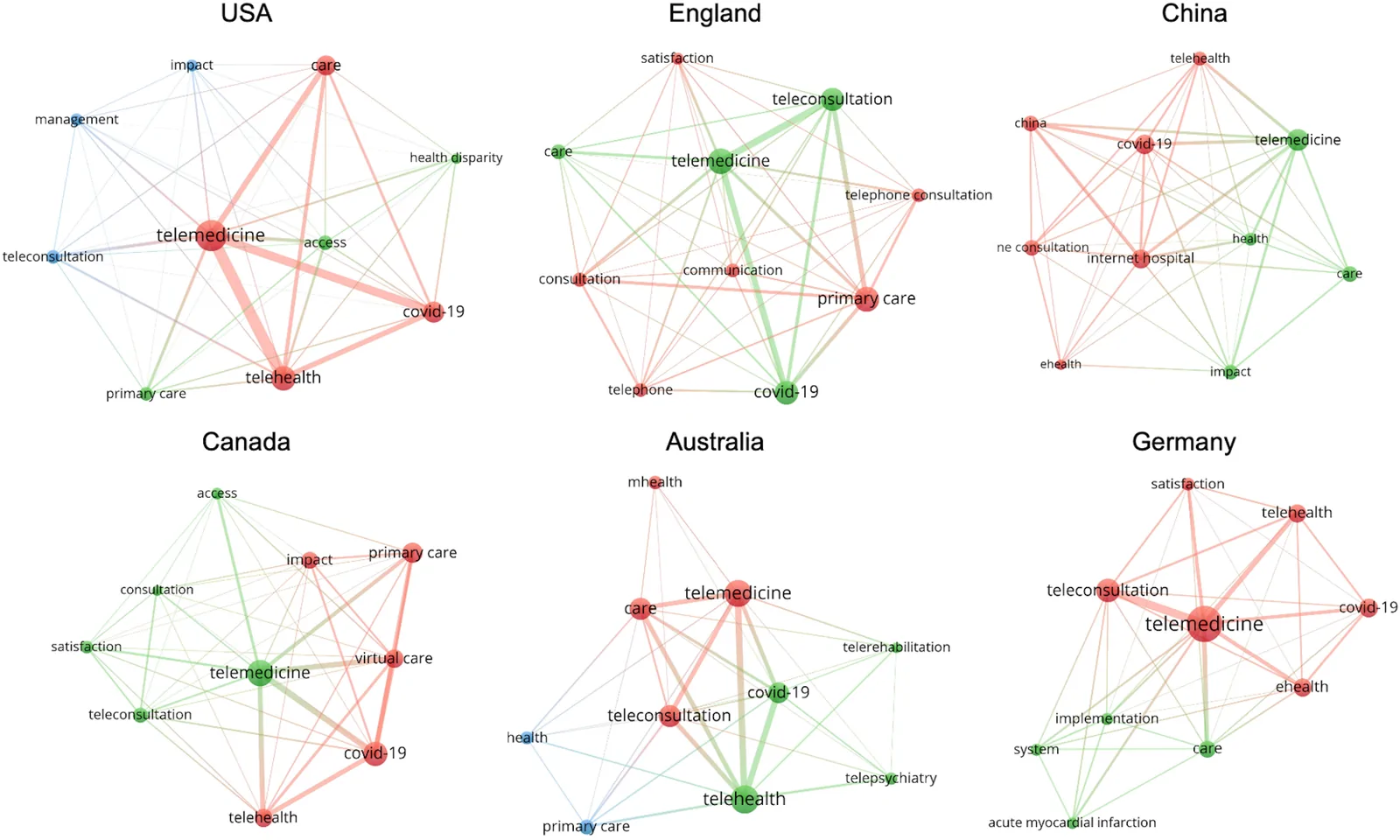
Country-specific keyword co-occurrence networks for the six most productive countries. Networks were constructed from country-filtered subsets of the final corpus using Keywords Plus. Association-strength normalization was applied to edge weights. Communities (clusters) were detected and color-coded. Node size represents keyword frequency, and edge width represents co-occurrence strength.

## Discussion

### Principal findings

Using the SCI-Expanded corpus, this study analyzed 2,426 publications from 1990 to 2025 and delineated the accelerated growth and structural reconfiguration of research on online consultations over the past 35 years. The time series pattern indicates a transition from a low-baseline exploratory phase to rapid expansion and, subsequently, a higher steady state. Specifically, output increased slowly from 1991 to 2013, reflecting a focus on conceptual development and feasibility-oriented methods; it then rose steadily during 2014–2019, consistent with technological maturation and the clarification of service models. Joinpoint regression identified two change points, in 2018 and 2022, indicating rapid expansion during 2018–2022 followed by post-peak adjustment after 2022. Although publication counts moderated after 2023, they remained markedly above pre-pandemic levels, suggesting normalization after scale adjustment rather than reversion to baseline. At the global level, scientific production was highly concentrated: the US and England occupied central positions in both output and collaboration networks. However, productivity and impact were not fully aligned, indicating cross-national differences in knowledge diffusion efficiency and research visibility. Thematically, countries shared a telemedicine-centered technical backbone but diverged in the post-pandemic period along institutionally shaped trajectories, the US increasingly emphasized access and equity, England remained anchored in primary care, China highlighted platform-based provision through internet hospitals, Canada reinforced the integration of virtual care with primary care, and Australia and Germany showed stronger specialization and implementation-oriented agendas. Signals from keyword and trend-topic analyses further suggest a shift in research emphasis from feasibility and service establishment toward management, quality, evaluation, and implementation, while informatics and engineering terms predominantly served enabling roles positioning online consultation as a digitally mediated service delivery model underpinned by platforms and interoperability. At the institutional level, highly cited and highly connected nodes clustered around medical schools and academic health systems, revealing a translationally oriented collaboration structure.

### Interpretation in context

The temporal pattern identified in this study coincided with the diffusion of digital infrastructure and policy incentives. The first marked upswing, around 2010, occurred alongside the broader uptake of electronic health records, patient portals, and secure messaging. These developments may have reduced transaction costs for asynchronous communication and follow-up and expanded the feasible use cases for online consultation. The rapid expansion during 2018–2022 also coincided with the COVID-19 shock and rapid policy responses, including expanded reimbursement and temporary relaxation of some service delivery constraints. These temporal associations are compatible with, but do not establish, infrastructure or policy effects. Notably, publication activity remained well above the pre-2020 baseline after the pandemic surge, suggesting a transition from emergency substitution to routinization within service delivery. Consistent with this shift, the thematic focus moved from feasibility and service set-up toward management, implementation, and evaluation.

### Gaps in institution level collaboration

While 100 countries have contributed to online consultation research, indicating broad global interest, the collaboration landscape remains distinctly “center-periphery.” A small number of high-output countries and institutions occupy the core of the network, with the US serving as the dominant hub with dense international ties. However, many other countries still have substantial room to expand and deepen cross-national collaboration. At the institutional level, collaboration is geographically clustered, with major blocs in North America, Europe-Australia, and comparatively limited connectivity among Asian nodes. This likely reflects regional differences in digital health infrastructure, health system organization, and research investment. Notably, cross-sector collaboration is structurally imbalanced, with university-hospital/health system partnerships being the strongest, while industry participation and network connectivity remain weaker, suggesting an underdeveloped interface between academic evidence generation and commercialization or system-scale implementation. This imbalance has two key implications. First, evidence may be disproportionately generated within large academic health systems and leading tertiary providers, potentially limiting external generalizability. Second, critical translation bottlenecks, such as platform iteration, interoperability, privacy compliance, and implementation at scale, may be insufficiently addressed due to limited cross-boundary coordination, slowing the conversion of research findings into scalable service models and policy tools. Closing this gap will likely require multinational, multi-institutional consortia, harmonized methods, and more open yet compliant data-sharing and collaboration mechanisms to lower barriers to cross-system comparisons.

### Hotspots and emerging trends in online consultation research

Across countries, the thematic backbone remains centered on telemedicine, consistently linked to COVID-19, care delivery, and primary care. However, in the post-pandemic period, the research frontier has increasingly focused on value realization. Specifically, the field is shifting (1) from supply-side expansion to evaluating effects and outcomes (e.g., impact, outcomes, quality, and satisfaction), (2) from general service delivery to platform- and pathway-based configurations (e.g., China’s internet hospital model), and (3) from generic telehealth applications to specialty and scenario-specific use cases (e.g., telepsychiatry in Australia and disease-context and implementation themes in Germany).

### Cross national differences and plausible drivers

To describe cross-country differences and their possible contexts (RQ3), we compared thematic networks across the six leading producer countries. The USA and England exhibited denser networks with more clusters; however, these structural differences may partly reflect their larger national corpora. China also showed multiple clusters, whereas Canada, Australia, and Germany displayed comparatively sparser networks. Despite these differences, high-frequency hubs converged on telemedicine, care delivery, and management, with divergence appearing in country-specific peripheral themes. England remained focused on primary care and health services, a pattern consistent with NHS England’s guidance on integrating online consultations into primary care service delivery [26]. Canada and Australia emphasized virtual care and specialty applications, while Germany retained disease-specific and implementation-related themes. China focused on internet hospitals, a pattern compatible with the broader policy context of Internet Plus Healthcare [27^,^^28^]. These policy-context connections provide a meaningful interpretive lens for understanding the observed keyword patterns, highlighting how national policy environments may shape thematic emphases in the literature.

Multi-country trend-topic lifespans exhibited an entry-expansion-reinforcement rhythm around three key inflection points (2011, 2018, and 2022). Around 2011, topics had longer active spans (Q1-Q3) but lower frequencies, consistent with early exploration and feasibility testing. By 2018, median years shifted rightward, and active periods became more concentrated, indicating institutionalization and scaling. After 2022, increased frequencies and more recent median years suggested a post-pandemic shift in emphasis from whether online consultation can be delivered to how it can be delivered better more equitably and sustainably.

### Strengths and limitations

To the best of our knowledge, this study is one of the first to provide a multidimensional bibliometric synthesis of online consultation research. Compared to previous work, we integrated multiple visualization tools and bibliometric techniques to generate finer descriptive statistics and provide deeper insights into temporal dynamics, collaboration structures, and thematic hotspots. We also performed additional trend validation and hotspot comparisons for leading countries with distinct health financing and system contexts, with the aim of informing future research and implementation.

Several limitations should be noted. First, although SCI-Expanded provides standardized cited-reference, affiliation, country/region, and keyword-related fields suitable for bibliometric mapping, reliance on this single index may under-represent publications indexed only in SSCI, ESCI, PubMed, Scopus, regional databases, or non-English databases. Second, the English-language and Article-only eligibility criteria may underestimate contributions published in other languages or in conference proceedings and other document types. Third, cross-country comparisons may be influenced by differences in country-specific corpus size; smaller national corpora can yield sparser keyword networks and fewer detectable thematic communities. Fourth, Jaccard similarity based on top keywords should be interpreted as a descriptive indicator rather than a comprehensive measure of thematic convergence. Future studies could integrate multiple databases and multilingual sources, and incorporate full-text semantic analysis, policy-practice data linkages, and real-world implementation outcomes to improve coverage and contextual interpretation.

### Policy implications and future work

Based on the observed bibliometric patterns and relevant policy literature, three tentative priorities may be considered for future empirical evaluation and policy development. First, payment and performance metrics could be aligned with the comparative advantages of online consultation by recognizing timely advice, asynchronous follow-up, and documented triage, thereby easing pressure on in person capacity while discouraging low value throughput. Second, strengthen interoperability and workflow integration by enabling routine exchange of encounter summaries, structured triage data, and prescribing within electronic health records to reduce duplication and improve safety [29^,^^30^]. Third, design for equity by addressing known barriers among older adults and digitally excluded populations, achieved through simplified authentication, caregiver proxy access, telephone fallback, and clear response time standards [31^,^^32^]. At the service level, dynamic cross specialty pooling, algorithm assisted routing, and capacity dashboards can mitigate the demand imbalances signaled by our keyword evidence.

Future work should examine whether and how reimbursement stability, privacy regimes, and interoperability readiness are associated with adoption trajectories and thematic portfolios across countries. Equity-oriented research should connect bibliometric signals to population-level outcomes to identify who benefits and who is left behind, with particular attention to older adults and low-connectivity regions [33]. Methodologically, cross-database and cross-language corpora, along with harmonized taxonomies for online consultation tasks and outcomes, would improve recall and comparability [34]. Additionally, real-world evaluations linking service design choices to clinical outcomes, costs, and clinician workload are needed to complement publication-based indicators [35].

## Conclusion

Online consultation research has evolved from early feasibility testing to a more mature agenda centered on management, implementation, and outcome evaluation. Publication activity declined after the 2022 peak but remained above the pre-pandemic baseline, suggesting post-peak adjustment rather than a return to the earlier low-output phase. Knowledge production and collaboration remain concentrated in a small set of countries and clinically oriented academic health systems, with the US and England occupying central network positions. However, productivity and impact are not fully aligned across countries. Thematic trajectories reveal a shared telemedicine-centred backbone that diversified during the pandemic and subsequently differentiated into institutionally shaped pathways. By integrating temporal trends, collaboration structures, and cross-country thematic evolution, this study clarifies where the field stands and highlights actionable levers for advancing both evidence and practice. Future progress will likely depend on sustained reimbursement and governance, interoperable and privacy-preserving data infrastructures, and equity-first service design and evaluation that explicitly include older adults and digitally marginalized populations.

## Supporting information

S1 Appendix Search strategy, filtering workflow, and supplementary database comparison. (DOCX)

S2 Appendix Keyword-merging dictionary and quantitative summary. (DOCX)

S3 Appendix Bibliometric tool integration strategy. (DOCX)

S4 Appendix Supplementary bibliometric descriptive tables. (DOCX)

S5 Appendix Joinpoint regression model selection and segmented estimates. (DOCX)

S6 Appendix Normalized country-specific keyword frequencies and supplementary Jaccard similarity analysis. (DOCX)

S1 Data Metadata-level analytical dataset of included publications. (ZIP)

## References

[1] Sugawara Y, Hirakawa Y, Iwagami M, Inokuchi R, Wakimizu R, Nangaku M. Metrics for Evaluating Telemedicine in Randomized Controlled Trials: Scoping Review. J Med Internet Res. 2025;27:e67929. doi: 10.2196/67929 39889298 PMC11829184

[2] Bonica GM, Johns RW, Jadvar H. Telehealth and telemedicine: regulatory and medicolegal landscape. Clin Nucl Med. 2024;49(7):644–7. 10.1097/RLU.0000000000005254 38769654

[3] Telematics W. H. O. Group Consultation on Health. A health telematics policy in support of WHO’s health-for-all strategy for global health development. Geneva: World Health Organization. 1998.

[4] Craig J, Patterson V. Introduction to the practice of telemedicine. J Telemed Telecare. 2005;11(1):3–9. doi: 10.1177/1357633X0501100102 15829036

[5] China The State Council General Office of the People’s Republic of. Opinions on promoting the development of “Internet Plus Healthcare. Beijing, China: The State Council. 2018.

[6] Omboni S, McManus RJ, Bosworth HB, Chappell LC, Green BB, Kario K, et al. Evidence and Recommendations on the Use of Telemedicine for the Management of Arterial Hypertension: An International Expert Position Paper. Hypertension. 2020;76(5):1368–83. doi: 10.1161/HYPERTENSIONAHA.120.15873 32921195

[7] Barbosa W, Zhou K, Waddell E, Myers T, Dorsey ER. Improving Access to Care: Telemedicine Across Medical Domains. Annu Rev Public Health. 2021;42:463–81. doi: 10.1146/annurev-publhealth-090519-093711 33798406

[8] European Parliamentary Research Service. The rise of digital health technologies during the pandemic.

[9] Qiu Y, Liu Y, Ren W, Qiu Y, Ren J. Internet-based and mobile-based general practice: cross-sectional survey. J Med Internet Res. 2018;20(9):e266. 10.2196/jmir.8378 PMC6300040 30257819

[10] Li J, Zhang Y, Ma L, Liu X. The Impact of the Internet on Health Consultation Market Concentration: An Econometric Analysis of Secondary Data. J Med Internet Res. 2016;18(10):e276. doi: 10.2196/jmir.6423 27793793 PMC5106558

[11] Chen J, Jie L, Su X, Zhang X, Guo D, Wang J, et al. COVID-19 Pandemic and the Operation of a Tertiary Hospital in Tianjin-The Impact of Internet Healthcare on Hospital Business Indicators. Front Public Health. 2022;10:892773. doi: 10.3389/fpubh.2022.892773 35669752 PMC9163548

[12] Martín-Martín A, Thelwall M, Orduna-Malea E, Delgado López-Cózar E. Google Scholar, Microsoft Academic, Scopus, Dimensions, Web of Science, and OpenCitations’ COCI: a multidisciplinary comparison of coverage via citations. Scientometrics. 2021;126(1):871–906. 10.1007/s11192-020-03690-4 PMC7505221 32981987

[13] Ivanitskaya LV, Zikos D, Erzikova E. Multidisciplinary contributions and research trends in eHealth scholarship (2000-2024): bibliometric analysis. J Med Internet Res. 2025;27(e60071). 10.2196/60071 PMC12209731 40522718

[14] Maugeri A, Barchitta M, Basile G, Agodi A. Public and Research Interest in Telemedicine From 2017 to 2022: Infodemiology Study of Google Trends Data and Bibliometric Analysis of Scientific Literature. J Med Internet Res. 2024;26:e50088. doi: 10.2196/50088 38753427 PMC11140276

[15] Almathami HKY, Win KT, Vlahu-Gjorgievska E. Barriers and Facilitators That Influence Telemedicine-Based, Real-Time, Online Consultation at Patients’ Homes: Systematic Literature Review. J Med Internet Res. 2020;22(2):e16407. doi: 10.2196/16407 32130131 PMC7059083

[16] Antonio S, Joseph D, Parsons J, Atherton H. Experiences of remote consultation in UK primary care for patients with mental health conditions: A systematic review. Digit Health. 2024;10:20552076241233969. doi: 10.1177/20552076241233969 38465292 PMC10924560

[17] Amerio A, Vai E, Bruno E, Costanza A, Escelsior A, Odone A, et al. COVID-19 Impact on the Italian Community-based System of Mental Health Care: Reflections and Lessons Learned for the Future. Clin Psychopharmacol Neurosci. 2023;21(1):2–9. doi: 10.9758/cpn.2023.21.1.2 36700307 PMC9889896

[18] Jiang H, Mi Z, Xu W. Online medical consultation service-oriented recommendations: systematic review. J Med Internet Res. 2024;26:e46073. 10.2196/46073 PMC11322685 38777810

[19] Campbell K, Greenfield G, Li E, O’Brien N, Hayhoe B, Beaney T, et al. The Impact of Virtual Consultations on the Quality of Primary Care: Systematic Review. J Med Internet Res. 2023;25:e48920. doi: 10.2196/48920 37647117 PMC10500356

[20] Page MJ, Moher D, Bossuyt PM, Boutron I, Hoffmann TC, Mulrow CD, et al. PRISMA 2020 explanation and elaboration: updated guidance and exemplars for reporting systematic reviews. BMJ. 2021;372:n160. doi: 10.1136/bmj.n160 33781993 PMC8005925

[21] Cobo MJ, López-Herrera AG, Herrera-Viedma E, Herrera F. Science mapping software tools: Review, analysis, and cooperative study among tools. J Am Soc Inf Sci. 2011;62(7):1382–402. doi: 10.1002/asi.21525

[22] Qi W, Zhu X, He D, Wang B, Cao S, Dong C, et al. Mapping Knowledge Landscapes and Emerging Trends in AI for Dementia Biomarkers: Bibliometric and Visualization Analysis. J Med Internet Res. 2024;26:e57830. doi: 10.2196/57830 39116438 PMC11342017

[23] van Eck NJ, Waltman L. VOSviewer manual 1.6.20. University of Leiden.

[24] Price DJ. Networks of scientific papers. Science. 1965;149(3683):510–5. 10.1126/science.149.3683.510 14325149

[25] Aria M, Cuccurullo C. bibliometrix: An R-tool for comprehensive science mapping analysis. Journal of Informetrics. 2017;11(4):959–75. doi: 10.1016/j.joi.2017.08.007

[26] England N. Using online consultations in primary care: implementation toolkit. NHS England. 2019.

[27] World Health Organization. Global strategy on digital health 2020-2025. Geneva: World Health Organization. 2021.

[28] Wakeman M, Buckman DW, El-Toukhy S. Disparities in Digital Health Care Use in 2022. JAMA Network Open. 2025;8(4):e255359-e. 10.1001/jamanetworkopen.2025.5359 PMC12550827 40244585

[29] Li E, Clarke J, Ashrafian H, Darzi A, Neves AL. The Impact of Electronic Health Record Interoperability on Safety and Quality of Care in High-Income Countries: Systematic Review. J Med Internet Res. 2022;24(9):e38144. doi: 10.2196/38144 36107486 PMC9523524

[30] Cahill M, Cleary BJ, Cullinan S. The influence of electronic health record design on usability and medication safety: systematic review. BMC Health Serv Res. 2025;25(1):31. doi: 10.1186/s12913-024-12060-2 39762805 PMC11705737

[31] Leff B, Ritchie CS, Rising KL, Cannon K, Wardlow L. Addressing barriers to equitable telehealth for older adults. Front Med (Lausanne). 2025;12:1483366. doi: 10.3389/fmed.2025.1483366 39917065 PMC11799546

[32] Mao A, Tam L, Xu A, Osborn K, Sheffrin M, Gould C, et al. Barriers to Telemedicine Video Visits for Older Adults in Independent Living Facilities: Mixed Methods Cross-sectional Needs Assessment. JMIR Aging. 2022;5(2):e34326. doi: 10.2196/34326 35438648 PMC9066341

[33] Lam K, Lu AD, Shi Y, Covinsky KE. Assessing Telemedicine Unreadiness Among Older Adults in the United States During the COVID-19 Pandemic. JAMA Intern Med. 2020;180(10):1389–91. doi: 10.1001/jamainternmed.2020.2671 32744593 PMC7400189

[34] Mongeon P, Paul-Hus A. The journal coverage of Web of Science and Scopus: a comparative analysis. Scientometrics. 2016;106(1):213–28.

[35] Darley S, Coulson T, Peek N, Moschogianis S, van der Veer SN, Wong DC, et al. Understanding How the Design and Implementation of Online Consultations Affect Primary Care Quality: Systematic Review of Evidence With Recommendations for Designers, Providers, and Researchers. J Med Internet Res. 2022;24(10):e37436. doi: 10.2196/37436 36279172 PMC9621309

