# Supplementary material for: Global trends and thematic evolution in online consultation with digital health technologies: Implications for future implementation and policy development
Source: PLOS Digit Health. 2026 Oct 29;5(10):e0001779. doi: 10.1371/journal.pdig.0001779 (PMC13718943; doi:10.1371/journal.pdig.0001779)
Supplement: Supplementary file 1 [file pdig.0001779.s001.docx]

**S1 Appendix. Search Strategy, Filtering Workflow, and Supplementary Database Comparison (SCI-Expanded, PubMed, and Scopus)**

**Table A. SCI-Expanded search strategy and retrieval results**

| **Set** | **Concept** | **Search query** | **Records returned (n)** |
| --- | --- | --- | --- |
| #1 | Actor block | TI=(patient* OR "primary care" OR clinic* OR outpatient OR physician* OR doctor* OR health* OR medic*) | 5,180,804 |
| #2 | Online consultation block | TS=("online consultation" OR "online visit*" OR "virtual visit*" OR "remote consultation" OR teleconsult* OR "e-visit*" OR "online triage" OR "digital triage" OR "telephone consult*" OR "phone consult*" OR "telemedicine visit*" OR "internet hospital*" OR "online hospital*") | 6,690 |
| #3 | Exclusion block | TS=(legal OR law OR marketing OR business OR "academic advising") | 1,060,081 |
| #4 | Combined search set | (#1 AND #2) NOT #3 | 3,173 |

**Note.** *TI, title; TS, topic. The TS field includes the title, abstract, author keywords, and Keywords Plus. The initial retrieval set was defined as (#1 AND #2) NOT #3.*

**Table B. Sequential filtering and final analytical corpus**

| **Step** | **Stage / filter** | **Records retained (n)** | **Records excluded (n)** | **Details** |
| --- | --- | --- | --- | --- |
| 1 | Initial retrieval: (#1 AND #2) NOT #3 | 3,173 | — | Search set before language and document-type filtering. |
| 2 | Language = English | 3,089 | 84 | Non-English records: German (29), French (25), Spanish (24), Portuguese (3), Hungarian (1), Icelandic (1), and Polish (1). |
| 3 | Document type = Article | 2,426 | 663 | Excluded labels included Review Article (226), Meeting Abstract (226), Editorial Material (69), Letter (53), Proceedings Paper (43), Early Access (37), News Item (6), Correction (4), Retracted Publication (3), and Book Chapter (1). |
| 4 | Final analytical corpus | 2,426 | — | English-language articles indexed in SCI-Expanded and included in the bibliometric analysis. |

**Note**: *Document-type labels were not mutually exclusive; therefore, the sum of excluded labels exceeds the number of excluded records.*

**Table C. Retrieval volume and DOI availability after date, language, and publication-type filters**

| **Database** | **Records retrieved (n)** | **Records with DOI (n)** | **DOI completeness (%)** | **Unique DOI (n)** | **Interpretation** |
| --- | --- | --- | --- | --- | --- |
| SCI-Expanded | 2,426 | 2,374 | 97.9 | 2,373 | Final analytical corpus. Structured cited-reference, affiliation, country/region, and keyword-related fields were compatible with the planned bibliometric workflow. |
| PubMed | 4,041 | 3,889 | 96.2 | 3,889 | Higher retrieval volume reflects broad biomedical and clinical coverage and non-equivalent publication-type indexing. PubMed publication-type tags are non-mutually exclusive. |
| Scopus | 6,979 | 6,583 | 94.3 | 6,579 | Largest retrieval set, consistent with broad multidisciplinary and regional journal coverage and use of the TITLE-ABS-KEY field. |

**Table D. Pairwise DOI overlap across databases**

| **Database comparison** | **Overlapping unique DOI (n)** | **Unique DOI in database A (n)** | **Unique DOI in database B (n)** | **Overlap of database A (%)** | **Overlap of database B (%)** | **Jaccard similarity (%)** |
| --- | --- | --- | --- | --- | --- | --- |
| SCI-Expanded vs PubMed | 2,160 | 2,373 | 3,889 | 91.0 | 55.5 | 52.7 |
| SCI-Expanded vs Scopus | 2,119 | 2,373 | 6,579 | 89.3 | 32.2 | 31.0 |
| PubMed vs Scopus | 3,082 | 3,889 | 6,579 | 79.2 | 46.8 | 41.7 |

**Table E. Distribution of unique DOI across database combinations**

| **DOI overlap category** | **Unique DOI (n)** |
| --- | --- |
| All three databases | 1,996 |
| SCI-Expanded + PubMed only | 164 |
| SCI-Expanded + Scopus only | 123 |
| PubMed + Scopus only | 1,086 |
| SCI-Expanded only | 90 |
| PubMed only | 643 |
| Scopus only | 3,374 |

**Note.** *DOI-based overlap was calculated using unique normalized DOI values. Records without a DOI were excluded from DOI-based overlap denominators. DOI normalization included lowercasing and removal of common prefixes and trailing punctuation.*

**Table F. Database-specific search strategies and retrieval results across SCI-Expanded, PubMed, and Scopus.**

| Database | Field Mapping | Search Strategy | Filters | Retrieved Records |
| --- | --- | --- | --- | --- |
| **SCI-Expanded (WoS Core Collection)** | TI = Title; TS = Topic (Title, Abstract, Author Keywords, Keywords Plus) | TI=(patient* OR "primary care" OR clinic* OR outpatient OR physician* OR doctor* OR health* OR medic*) AND TS=("online consultation" OR "online visit*" OR "virtual visit*" OR "remote consultation" OR teleconsult* OR "e-visit*" OR "online triage" OR "digital triage" OR "telephone consult*" OR "phone consult*" OR "telemedicine visit*" OR "internet hospital*" OR "online hospital*") NOT TS=(legal OR law OR marketing OR business OR "academic advising") | 1990–2025; English; Article | 2,426 |
| **PubMed** | WoS TI → [Title]; WoS TS → [Title/Abstract] | (patient*[Title] OR "primary care"[Title] OR clinic*[Title] OR outpatient*[Title] OR physician*[Title] OR doctor*[Title] OR health*[Title] OR medic*[Title]) AND ("online consultation"[Title/Abstract] OR "online consultations"[Title/Abstract] OR "online visit"[Title/Abstract] OR "virtual visit"[Title/Abstract] OR "remote consultation"[Title/Abstract] OR teleconsult* OR "e-visit" OR "online triage" OR "telephone consultation" OR "telemedicine visit" OR "internet hospital") NOT (legal OR law OR marketing OR business OR "academic advising") | 1990–2025; English; Journal Article | 4,041 |
| **Scopus** | TITLE / TITLE-ABS-KEY | TITLE(patient* OR "primary care" OR clinic* OR outpatient* OR physician* OR doctor* OR health* OR medic*) AND TITLE-ABS-KEY("online consultation" OR "virtual visit*" OR "remote consultation" OR teleconsult* OR "e-visit*" OR "online triage" OR "telephone consult*" OR "telemedicine visit*" OR "internet hospital*" OR "online hospital*") AND NOT TITLE-ABS-KEY(legal OR law OR marketing OR business OR "academic advising") | 1990–2025; English; Article | 6,979 |

**Note**: *Search terms were adapted to database-specific field tags and syntax while preserving the same conceptual blocks.*
