# Supplementary material for: Global trends and thematic evolution in online consultation with digital health technologies: Implications for future implementation and policy development
Source: PLOS Digit Health. 2026 Oct 29;5(10):e0001779. doi: 10.1371/journal.pdig.0001779 (PMC13718943; doi:10.1371/journal.pdig.0001779)
Supplement: Supplementary file 2 [file pdig.0001779.s002.docx]

**S2 Appendix. Keyword-merging dictionary and quantitative summary**

**Table A. Quantitative summary of keyword consolidation**

| **Item** | **Value** |
| --- | --- |
| Original keyword labels before consolidation | 554 |
| Total keyword occurrences before consolidation | 11,329 |
| Variant labels mapped to standardized terms | 151 |
| Keyword occurrences affected by mapping | 2,522 |
| Proportion of all keyword occurrences affected | 22.3% |
| Standardized keyword labels after consolidation | 472 |
| Reduction in unique keyword labels | 82 |
| Percentage reduction in unique keyword labels | 14.8% |

**Table B. Selected high-frequency keyword labels before consolidation**

| **Keyword label** | **Occurrences** | **Cluster** | **Links** | **Total link strength** |
| --- | --- | --- | --- | --- |
| telemedicine | 995 | 6 | 520 | 4725 |
| covid-19 | 497 | 4 | 426 | 2242 |
| telehealth | 459 | 4 | 446 | 2456 |
| care | 353 | 6 | 423 | 1840 |
| remote consultation | 191 | 8 | 313 | 1055 |
| management | 170 | 1 | 327 | 992 |
| impact | 168 | 3 | 334 | 971 |
| teleconsultation | 165 | 5 | 277 | 866 |
| primary care | 132 | 2 | 261 | 702 |
| satisfaction | 126 | 10 | 259 | 767 |

**Table C. Sensitivity comparison of keyword clustering before and after consolidation**

| **Comparison item** | **Before consolidation** | **After consolidation and comparison** |
| --- | --- | --- |
| Unique keyword labels, n | 554 | 472; decreased by 82 labels (14.8%) |
| Number of clusters, n | 12 | 13; a small change indicating limited redistribution of cluster boundaries |
| Top-10 keyword overlap | Original top-10 set | Nine of the top 10 keywords were shared (90%). Remote consultation was consolidated into teleconsultation, while access entered the top-10 set. |
| Major thematic areas | Telemedicine/telehealth; COVID-19; primary care and service delivery; management and outcomes; satisfaction and technology adoption; mental health and specialty applications | The same broad thematic areas remained visible after consolidation. |

**Note**: *Both analyses used identical VOSviewer settings (minimum occurrence = 5; association-strength normalization; resolution = 1.00; minimum cluster size = 1; merge small clusters enabled).*

**Table D. Keyword-merging dictionary**

| No | Keywords | Merged Keywords |
| --- | --- | --- |
| 1 | access | health services accessibility |
|  |  | healthcare access |
| 2 | acute myocardial infarction | acute myocardial-infarction |
| 3 | adolescent | adolescents |
| 4 | adult | adults |
| 5 | after hours care | after-hours care |
| 6 | blood pressure | blood-pressure |
| 7 | cardiovascular disease | cardiovascular-disease |
| 8 | caregiver | caregivers |
| 9 | center | centers |
| 10 | child | children |
| 11 | chronic obstructive pulmonary disease | copd |
| 12 |  | obstructive pulmonary-disease |
| 13 | community | communities |
| 14 | consultation | consultations |
| 15 | cost | costs |
| 16 | country | countries |
| 17 | covid-19 | coronavirus |
|  |  | coronavirus disease 2019 |
|  |  | covid |
|  |  | covid-19 pandemic |
| 18 | criterion | criteria |
| 19 | cross sectional study | cross-sectional studies |
| 20 | decision making | decision-making |
| 21 | determinant | determinants |
| 22 | developing country | developing countries |
|  |  | developing-countries |
| 23 | diabetes mellitus | diabetes |
| 24 | diagnostic accuracy | diagnostic-accuracy |
| 25 | disorder | disorders |
| 26 | doctor | doctors |
| 27 | double blind | double-blind |
| 28 | ehealth | e-health |
| 29 | electronic health record | electronic health records |
| 30 | email | e-mail |
|  |  | electronic mail |
| 31 | email communication | e-mail communication |
| 32 | emergency department | emergency-department |
| 33 | error | errors |
| 34 | european society | european-society |
| 35 | e-visit | e-visits |
|  |  | electronic visits |
| 36 | experience | experiences |
| 37 | follow up | follow-up |
| 38 | guideline | guidelines |
| 39 | health care | health-care |
|  |  | healthcare |
| 40 | health care delivery | service delivery |
| 41 | health care professional | healthcare professionals |
| 42 | health care utilization | healthcare utilization |
| 43 | health disparity | disparities |
|  |  | healthcare disparities |
| 44 | health service | health services |
| 45 | heart failure | heart-failure |
| 46 | hiv | hiv/aids |
| 47 | hospital | hospitals |
| 48 | hour primary care | hours primary-care |
| 49 | image | images |
| 50 | individual | individuals |
| 51 | information seeking | information-seeking |
| 52 | information technology | information-technology |
| 53 | injury | injuries |
| 54 | intervention | interventions |
| 55 | interview | interviews |
| 56 | lesson | lessons |
| 57 | mhealth | m-health |
|  |  | mobile health |
| 58 | mixed method | mixed methods |
| 59 | model | models |
| 60 | need | needs |
| 61 | nurse | nurses |
| 62 | older adult | older adults |
|  |  | older-adults |
| 63 | online consultation | e-consultation |
|  |  | online medical consultation |
| 64 | opportunity | opportunities |
| 65 | out of hour | out-of-hours |
| 66 | outcome | outcomes |
| 67 | outpatient | outpatients |
| 68 | pandemic | pandemics |
| 69 | parent | parents |
| 70 | patient | patients |
| 71 | patient centered care | patient-centered care |
| 72 | patient preference | patient preferences |
| 73 | patient-reported outcome measure | patient-reported outcome measures |
| 74 | pattern | patterns |
| 75 | pediatric | pediatrics |
| 76 | perception | perceptions |
| 77 | perspective | perspectives |
| 78 | pharmacist | pharmacists |
| 79 | physical activity | physical-activity |
| 80 | physician | physicians |
| 81 | physician patient relation | physician-patient relations |
| 82 | practitioner | practitioners |
| 83 | predictor | predictors |
| 84 | primary care | family medicine |
|  |  | family practice |
|  |  | general practice |
|  |  | general-practice |
|  |  | primary health care |
|  |  | primary healthcare |
|  |  | primary-care |
| 85 | primary care physician | family physicians |
|  |  | general practitioner |
|  |  | general practitioners |
|  |  | general-practitioner |
|  |  | general-practitioners |
|  |  | primary care physicians |
|  |  | primary-care physicians |
| 86 | provider | providers |
| 87 | quality of care | quality-of-care |
| 88 | quality of life | quality-of-life |
| 89 | randomized controlled trial | randomised controlled trial |
|  |  | randomized controlled-trial |
| 90 | recommendation | recommendations |
| 91 | referral | referrals |
| 92 | retrospective study | retrospective studies |
| 93 | risk factor | risk-factors |
| 94 | rural health service | rural health services |
| 95 | same day appointment | same-day appointments |
| 96 | self care | self-care |
| 97 | self efficacy | self-efficacy |
| 98 | self management | self-management |
| 99 | service | services |
| 100 | shared decision making | shared decision-making |
| 101 | skill | skills |
| 102 | strategy | strategies |
| 103 | survey and questionnaire | surveys and questionnaires |
| 104 | survivor | survivors |
| 105 | symptom | symptoms |
| 106 | system | systems |
| 107 | telecommunication | telecommunications |
| 108 | teleconsultation | joint teleconsultations |
|  |  | remote consultation |
|  |  | remote consultations |
|  |  | teleconsult |
|  |  | teleconsultations |
|  |  | teleconsulting |
| 109 | telehealth | tele-health |
| 110 | telemedicine | tele-medicine |
| 111 | telephone consultation | telephone consultations |
| 112 | tissue plasminogen activator | tissue-plasminogen activator |
| 113 | trend | trends |
| 114 | united states | united-states |
| 115 | veteran | veterans |
| 116 | video visit | video visits |
| 117 | virtual visit | virtual visits |
| 118 | visit | visits |
| 119 | vulnerable population | vulnerable populations |
| 120 | woman | women |
| 121 | worker | workers |
