# Supplementary material for: Global trends and thematic evolution in online consultation with digital health technologies: Implications for future implementation and policy development
Source: PLOS Digit Health. 2026 Oct 29;5(10):e0001779. doi: 10.1371/journal.pdig.0001779 (PMC13718943; doi:10.1371/journal.pdig.0001779)
Supplement: Supplementary file 3 [file pdig.0001779.s003.docx]

**S3 Appendix. Bibliometric tool integration strategy.**

**Table A. Overview of the bibliometric tool integration strategy.**

| **Tools**  **Functions** | **VOSviewer** | **Bibliometrix** | **Gephi** | **Cortext** | **Joinpoint** | **gCLUTO** | **R-studio** | **Echarts** |
| --- | --- | --- | --- | --- | --- | --- | --- | --- |
| Output |  |  |  |  | **√** |  |  |  |
| Author | **√** |  | **√** |  |  |  |  | **√** |
| Organization | **√** |  |  |  |  |  | **√** | **√** |
| Country | **√** |  |  |  |  |  |  | **√** |
| Keyword Theme |  | **√** |  |  |  |  |  |  |
| Keyword Progress |  | **√** |  |  |  |  |  |  |
| Keyword Cluster |  |  |  |  |  | **√** |  |  |
| Grant |  |  |  | **√** |  |  |  |  |
| Disciplinary |  |  |  | **√** |  |  |  |  |
| Highly Cited Research |  | **√** |  |  |  |  |  |  |
| Journal | **√** |  |  |  |  |  |  |  |
