# Supplementary material for: Global trends and thematic evolution in online consultation with digital health technologies: Implications for future implementation and policy development
Source: PLOS Digit Health. 2026 Oct 29;5(10):e0001779. doi: 10.1371/journal.pdig.0001779 (PMC13718943; doi:10.1371/journal.pdig.0001779)
Supplement: Supplementary file 4 [file pdig.0001779.s004.docx]

**S4 Appendix. Supplementary bibliometric descriptive tables**

**Table A. Top 10 institutions by publication output**

| ID | Institution | Full institution name | Country/Region | Documents | Citations | Total link strength (TLS) |
| --- | --- | --- | --- | --- | --- | --- |
| 1 | harvard med sch | Harvard Medical School | United States | 76 | 1586 | 8216 |
| 2 | univ toronto | University of Toronto | Canada | 49 | 1258 | 12725 |
| 3 | univ calif san francisco | University of California, San Francisco | United States | 45 | 745 | 4443 |
| 4 | univ oxford | University of Oxford | England | 36 | 942 | 5966 |
| 5 | univ penn | University of Pennsylvania | United States | 33 | 1543 | 3227 |
| 6 | mayo clin | Mayo Clinic | United States | 32 | 530 | 2076 |
| 7 | univ pittsburgh | University of Pittsburgh | United States | 32 | 707 | 4849 |
| 8 | johns hopkins univ | Johns Hopkins University | United States | 29 | 815 | 3501 |
| 9 | univ michigan | University of Michigan | United States | 28 | 805 | 2410 |
| 10 | univ washington | University of Washington | United States | 27 | 391 | 3159 |

**Table B. Top 10 authors by publication output.**

| Author | documents | citations | Average Citations | H-index | Institution | Country |
| --- | --- | --- | --- | --- | --- | --- |
| huang, jie | 16 | 448 | 28.00 | 7 | Kaiser Permanente | USA |
| christensen, morten bondo | 11 | 170 | 15.45 | 7 | [Aarhus University](https://ror.org/01aj84f44) | Denmark |
| huibers, linda | 10 | 160 | 16.00 | 6 | Aarhus University | Denmark |
| moth, grete | 10 | 191 | 19.10 | 8 | Aarhus University | Denmark |
| reed, mary e. | 10 | 335 | 33.50 | 4 | Kaiser Permanente | USA |
| rossaint, rolf | 10 | 335 | 33.50 | 10 | University Hospital Aachen | Germany |
| gopalan, anjali | 9 | 57 | 6.33 | 4 | Kaiser Permanente | USA |
| salisbury, chris | 9 | 1052 | 116.89 | 8 | City, University of London | United Kingdom |
| vedsted, peter | 9 | 164 | 18.22 | 7 | [Aarhus University](https://ror.org/01aj84f44) | Denmark |
| atherton, helen | 8 | 509 | 63.63 | 5 | University of Southampton | United Kingdom |

**Note:** *The h-index was calculated within the retrieved analytical corpus.*
