# Supplementary material for: Global trends and thematic evolution in online consultation with digital health technologies: Implications for future implementation and policy development
Source: PLOS Digit Health. 2026 Oct 29;5(10):e0001779. doi: 10.1371/journal.pdig.0001779 (PMC13718943; doi:10.1371/journal.pdig.0001779)
Supplement: Supplementary file 5 [file pdig.0001779.s005.docx]

**S5 Appendix. Joinpoint regression model selection and segmented estimates.**

**Table A. Model selection statistics**

| **Joinpoints** | **Observations** | **Parameters** | **DF** | **SSE** | **BIC** | **WBIC** |
| --- | --- | --- | --- | --- | --- | --- |
| 0 | 35 | 2 | 33 | 173496.27 | 8.7117 | 8.7117 |
| 1 | 35 | 4 | 31 | 58527.62 | 7.8282 | 7.8955 |
| 2 | 35 | 6 | 29 | 10359.43 | 6.2998 | 6.4878 |

**Table B. Joinpoint locations and segmented slopes**

| Segment | Years | Joinpoint | 95% CI | Slope | SE | P value |
| --- | --- | --- | --- | --- | --- | --- |
| 1 | 1991–2018 | — | — | 1.87 | 0.17 | <0.001 |
| 2 | 2018–2022 | 2018 | 2017–2019 | 82.99 | 4.81 | <0.001 |
| 3 | 2022–2025 | 2022 | 2022–2022 | −49.83 | 4.81 | <0.001 |

**Note**: *Joinpoint regression was fitted to annual publication counts using a linear segmented model.*
