# Supplementary material for: Global trends and thematic evolution in online consultation with digital health technologies: Implications for future implementation and policy development
Source: PLOS Digit Health. 2026 Oct 29;5(10):e0001779. doi: 10.1371/journal.pdig.0001779 (PMC13718943; doi:10.1371/journal.pdig.0001779)
Supplement: Supplementary file 6 [file pdig.0001779.s006.docx]

**S6 Appendix. Normalized country-specific keyword frequencies and supplementary Jaccard similarity analysis**

**Table A6.1. Top 10 standardized keywords after normalization**

| **Country** | **Records, n** | **Keyword** | **Raw occurrences** | **Total link strength** | **Normalized frequency per 100 records** |
| --- | --- | --- | --- | --- | --- |
| US | 891 | telemedicine | 430 | 652 | 48.26 |
|  |  | telehealth | 259 | 479 | 29.07 |
|  |  | covid-19 | 198 | 361 | 22.22 |
|  |  | care | 171 | 271 | 19.19 |
|  |  | access | 91 | 195 | 10.21 |
|  |  | primary care | 81 | 146 | 9.09 |
|  |  | teleconsultation | 79 | 152 | 8.87 |
|  |  | management | 66 | 101 | 7.41 |
|  |  | impact | 61 | 96 | 6.85 |
|  |  | health disparity | 50 | 117 | 5.61 |
| England | 301 | telemedicine | 69 | 104 | 22.92 |
|  |  | primary care | 68 | 80 | 22.59 |
|  |  | covid-19 | 61 | 73 | 20.27 |
|  |  | teleconsultation | 58 | 79 | 19.27 |
|  |  | care | 25 | 37 | 8.31 |
|  |  | consultation | 23 | 51 | 7.64 |
|  |  | communication | 20 | 35 | 6.64 |
|  |  | telephone | 20 | 42 | 6.64 |
|  |  | telephone consultation | 20 | 35 | 6.64 |
|  |  | satisfaction | 16 | 34 | 5.32 |
| China | 176 | telemedicine | 40 | 49 | 22.73 |
|  |  | covid-19 | 31 | 43 | 17.61 |
|  |  | internet hospital | 30 | 43 | 17.05 |
|  |  | care | 21 | 23 | 11.93 |
|  |  | china | 20 | 35 | 11.36 |
|  |  | online consultation | 20 | 26 | 11.36 |
|  |  | impact | 18 | 25 | 10.23 |
|  |  | telehealth | 17 | 30 | 9.66 |
|  |  | health | 14 | 27 | 7.95 |
|  |  | ehealth | 10 | 19 | 5.68 |
| Canada | 150 | telemedicine | 55 | 93 | 36.67 |
|  |  | covid-19 | 47 | 82 | 31.33 |
|  |  | primary care | 32 | 51 | 21.33 |
|  |  | telehealth | 27 | 55 | 18.0 |
|  |  | virtual care | 27 | 66 | 18.0 |
|  |  | impact | 23 | 31 | 15.33 |
|  |  | teleconsultation | 18 | 32 | 12.0 |
|  |  | satisfaction | 13 | 27 | 8.67 |
|  |  | access | 11 | 24 | 7.33 |
|  |  | consultation | 9 | 25 | 6.0 |
| Australia | 128 | telehealth | 37 | 63 | 28.91 |
|  |  | telemedicine | 34 | 53 | 26.56 |
|  |  | care | 23 | 39 | 17.97 |
|  |  | teleconsultation | 23 | 36 | 17.97 |
|  |  | covid-19 | 21 | 41 | 16.41 |
|  |  | primary care | 10 | 17 | 7.81 |
|  |  | mhealth | 9 | 6 | 7.03 |
|  |  | health | 8 | 12 | 6.25 |
|  |  | telepsychiatry | 7 | 13 | 5.47 |
|  |  | telerehabilitation | 5 | 12 | 3.91 |
| Germany | 102 | telemedicine | 56 | 90 | 54.9 |
|  |  | teleconsultation | 26 | 52 | 25.49 |
|  |  | covid-19 | 17 | 28 | 16.67 |
|  |  | ehealth | 16 | 33 | 15.69 |
|  |  | telehealth | 16 | 35 | 15.69 |
|  |  | care | 14 | 35 | 13.73 |
|  |  | satisfaction | 9 | 24 | 8.82 |
|  |  | implementation | 8 | 24 | 7.84 |
|  |  | system | 8 | 21 | 7.84 |
|  |  | acute myocardial infarction | 7 | 20 | 6.86 |

**Fig A. Pairwise Jaccard similarity matrix based on the top 15 standardized keywords for each country.**
